# Case Report: Takotsubo cardiomyopathy caused by acute surgical pathology against the background of colorectal cancer

**DOI:** 10.3389/fcvm.2025.1586353

**Published:** 2025-08-04

**Authors:** Vera Potievskaya, Victoria Khoronenko, Elena Kononova, Elena Paderina, Zaki Fashafsha, Andrey Kaprin

**Affiliations:** ^1^ P. Hertsen Moscow Oncology Research Institute—Branch or the Federal State Budgetary Institution “National Medical Research Radiological Centre” of the Ministry of Health of the Russian Federation, Moscow, Russia; ^2^ Russian Medical Academy of Postgraduate Education of Federal Scientific and Clinical Center for Specialized Medical Assistance and Medical Technologies of the Federal Medical Biological Agency, Moscow, Russia; ^3^ Peoples’ Friendship University of Russia, Moscow, Russia; ^4^ Institute for Personalized Cardiology, I.M. Sechenov First Moscow State Medical University (Sechenov University), Moscow, Russia

**Keywords:** Takotsubo syndrome, acute heart failure, perioperative period, postoperative complications, sigmoid colon cancer

## Abstract

We present a case of Takotsubo cardiomyopathy (TCM) in a young woman who has developed necrosis and perforation of the sigmoid intestine with a spontaneous opening of the abscess in the abdominal cavity, which caused purulent peritonitis. The patient underwent emergency surgical intervention. The postoperative period was complicated by a clinic of acute respiratory failure and bilateral pneumonia. In five days after surgery, the patient developed laboratory and instrumental signs of acute myocardial infarction. Changes similar to myocardial infarction appeared on electrocardiogram (ECG). Echocardiography revealed akinesis of the apical segments of the left ventricle and the ejection fraction of the left ventricle was reduced (LVEF) from 63 to 31%–35%. The level of NT—proBNP has increased significantly, but the level of troponin I increased to a lesser extent. Coronary angiography demonstrated normal arteries without any obstruction. As a result, a diagnosis Takotsubo cardiomyopathy caused by acute surgical pathology was made. In the future, the patient was discharged in a stable state. Subsequent echocardiography showed normalization of the systolic function of the left ventricle, the disappearance of ECG changes, and a decrease in the level of specific cardiomarkers.

## Introduction

Тakotsubo cardiomyopathy (TCM) (also apical cylinder syndrome, Acute stress-induced cardiomyopathy and “ broken heart syndrome”) is an acute clinical syndrome that often simulates the manifestations of the left ventricle front wall acute infarction (1). The term syndrome was first introduced when Sato and others (2). Published their report on five cases in the Japanese medical textbook in 1990. The first case of TCM from this series was treated in 1983 in the city hospital of Hiroshima. A 64-year-old woman addressed an acute chest pain corresponding to the acute myocardial infarction (AMI), typical changes in the electrocardiogram (ECG), but normal coronary arteries and an unusual species of left ventricle with a narrow neck and apical ballooning during systole. It is interesting that the pronounced abnormalities of the movement of the wall on the left ventriculography disappeared after 2 weeks.

Over time, TCM was more often diagnosed in Japan. Therefore, it was initially assumed that this condition exclusively affected individuals of Asian descent, as Takotsubo cardiomyopathy (TCM) was entirely unknown in the Western world until the first cases were reported by French and American research groups in the late 1990s (3, 4).

Desmet and others (5) presented the first series of disease cases in patients with the Caucasian race, using the term “Takotsubo”. Takotsubo Cardiomyopathy accounts for 2%—3% of patients with symptoms indicating acute coronary syndrome (6).

The exact pathogenesis of the Takotsubo Cardiomyopathy is not entirely clear, but literature data evidence that sympathetic stimulation playing the main role of it occurrence. In most cases, the syndrome is triggered by a specific emotional or physical trigger, and Тakotsubo syndrome (TTS) syndrome has been associated with conditions associated with excess catecholamines, such as pheochromocytoma (7).

However, potential pathophysiological mechanisms that may lead to increased sympathetic stimulation are diverse. One potential trigger for the activation of sympathetic stimulation is the rupture of a coronary plaque that may cause myocardial transient ischemia and myocardial stunning (8).

According to world literature, endothelial dysfunction is common in patients with TTS and may be a potential pathogenic mechanism for TTS. This may explain the tendency for epicardial and/or microvascular coronary artery spasm. The theory behind this concept is based on the imbalance between vasoconstrictive and vasodilative factors, which may serve as a crucial connection between stress and cardiac dysfunction in TTS (9).

Nevertheless, TTS doesn't affect everyone, and there are theories about the potential role of hormonal imbalance and genetic factors.

Based on the published literature about 90% of TTS patients are women with a mean age of 67–70 years. Women older than 55 years have a five-fold greater risk of developing TTS than women younger than 55 years and a 10-fold greater risk than men (7, 10).

It is possible that a decrease in estrogen levels after menopause may increase a woman's susceptibility to TTS (11).

A genetic predisposition may be influenced by environmental factors, the combined effects of multiple genes, or the presence of recessive susceptibility alleles. Variations in adrenergic genes can impact the functioning of receptors and subsequent signaling, suggesting that their prevalence might vary among individuals with TTS (12).

Functional variants of adrenergic receptor genes were associated with the magnitude of cardiac dysfunction in patients with subarachnoid hemorrhage and pheochromocytoma, conditions that can cause TTS syndrome. Variants of β1-adrenergic receptors and β2-adrenergic receptors were associated with a greater release of troponin I, and deletion of α2-adrenergic receptors was associated with a reduced left ventricular ejection fraction (1).

Cardiovascular diseases and cancer are two major causes of mortality in worldwide. They are closely related, notwithstanding the role of cancer in the pathogenesis of cardiovascular disorders, cancer treatment can often lead to cardiovascular complications, regardless of whether the treatment is surgery, chemotherapy, or radiation (13). And the development of Takotsubo syndrome isn't an exception.

The presence of cancer undoubtedly affects the emotional and psychological state of the patient and causes several physical pathological changes in the body, including oxidative stress and metabolic disorders. These changes can be a trigger for the development of TTS (14).

It has been found that several antitumor drugs are associated with TTS. Among these drugs, 5-fluorouracil (15, 16), which is part of two chemotherapy regimens FOLFOX, FOLFIRI. It has been observed that using of these regimens can potentially cause TTS (14).

Another chemotherapeutic drug that can potentially cause TTS is Bevacizumab (17). Bevacizumab is a monoclonal antibody whose antitumor activity depends on the inhibition of vascular endothelial growth factor (VEGF) signaling (18). Bevacizumab has been associated with many cardiovascular adverse events, especially arterial thrombosis (19).

However, patients with TCM usually have a favorable prognosis, and almost complete restoration of contractile function of the myocardium usually occurs 4–8 weeks after the appearance of symptoms in 96% of cases (20).

## Case presentation

### Patient K., 39 years old

Oncological diagnosis: Rectal cancer. Anterior resection of the rectum performed on August 30, 2018. Disease progression in April 2019 (liver metastases). Radiofrequency ablation of the lesion in segment IV of the liver performed on April 16, 2019. Disease progression in March 2023 (metastases to the lungs and brain). Resection of a tumor in the left frontal lobe of the brain performed on May 24, 2023. Seven cycles of polychemotherapy (PCT) with the FOLFOX [5-fluorouracil (5-FU)+ Oxaliplatin] + Bevacizumab regimen from August 2023 to November 2023. Disease progression in February 2024 (metastases to the left temporal region). Three cycles of PCT with the Irinotecan + Capecitabine regimen from June 2024 to August 2024. Disease progression in August 2024 (growth of brain and lung metastases). pT3N0M0, Stage IIA, Clinical Group II.

Cardiac diagnosis: No known cardiovascular pathology. At the P. Hertsen Moscow Oncology Research Institute (MORI)—the branch of the FSBI “National Medical Research Radiological Centre” (NMRRC) of the Ministry of Health of the Russian Federation, in the neurosurgery department, hospitalization of the patient was scheduled for October 24, 2024 for the removal of a metastatic lesion in the left parietal region. However, upon admission, the patient presented with complaints of diffuse abdominal pain (Table 1).

**Table 1:** Summary table with dates, clinical events, interventions, and diagnostic findings.

Event\intervention date	clinical events, interventions, and diagnostic findings
On October 24, 2024	A diffuse abdominal pain. Necrosis of the sigmoid colon with a contained perforation into the mesentery of the sigmoid colon, abscess formation, spontaneous rupture of the abscess into the abdominal cavity, and diffuse purulent peritonitis were diagnosed
On October 24, 2024	A laparotomy, exploration of the abdominal organs, obstructive resection of the rectum, formation of a descending colostomy, and lavage and drainage of the abdominal cavity were performed.
On October 25−26, 2024	A peritonitis, hypostatic pneumonia, and hydropneumothorax.
On October 25–26, 2024	A septic condition, hemoperfusion. A hydrothorax, pleural drainage
On October 27, 2024	An acute respiratory failure, Non-invasive ventilation, bilateral pneumonia
On October 28, 2024	On the electrocardiogram ECG inversion of the T wave in leads I, II, aVF, aVL, and V2-V6, as well as ST-segment depression in leads V5-V6 up to 1 mm. The QTc interval was 478 ms
On October 28, 2024	According to the echocardiography there was a reduction in left ventricular ejection fraction (LVEF) to 31–35% by Simpson's method. Akinesis of the anteroseptal segments at the mid and apical levels, as well as the posterolateral apical segments, was observed. Hypokinesis of the anteroseptal basal segments and the posterolateral mid segments of the left ventricle was also noted. Increase in NT-proBNP levels to 21,779 pg/ml and troponin I levels to 129.4 ng/L (reference values <100 ng/L).
On November 3, 2024	The diagnostic coronary angiography—no signs of coronary artery disease
On November 21, 2024	On the ECG—positive dynamics were noted: the ST segment returned to the isoelectric line, and the T wave became positive/ A decrease in NT-proBNP levels from 27,779 to 2,794 pg/ml and in troponin I levels from 129 to 22.9 ng/L was observed.
On November 26, 2024	On the echocardiography—the LVEF recovered to 51% by Simpson's method, with persistent mild hypokinesis of the anterior segment at the apical level and the anteroseptal segments at the basal and mid levels.

On October 24, 2024, x-ray of abdominal organs: signs of a perforated intestine were detected.

On October 24, 2024, after a laparotomy, exploration of the abdominal organs a necrosis of the sigmoid colon with a contained perforation into the mesentery of the sigmoid colon, abscess formation, spontaneous rupture of the abscess into the abdominal cavity, and diffuse purulent peritonitis were diagnosed. Obstructive resection of the rectum, formation of a descending colostomy, and lavage and drainage of the abdominal cavity were performed.

Following the surgical procedure, the patient was transferred to the intensive care unit with blood pressure (BP) 80/55 mmHg, heart rate 110 per minute, SpO2 is 95%. Vasopressor support with norepinephrine at a dose of 0.3–0.4 mcg/kg/min was administered for two days.

The postoperative period was complicated by the development of peritonitis, hypostatic pneumonia, and hydropneumothorax. On October 27, 2024, clinical signs of acute respiratory failure developed: oxygen saturation decreased to 80%–85% despite oxygen insufflation at a flow rate of 5–7 L/min. Non-invasive ventilation (CPAP/BIPAP) with an FIO2 of 75%–80% was initiated. According to the X -ray of the chest organs, bilateral pneumonia was identified. The following day, the patient was transitioned to spontaneous breathing.

Due to septic condition, three courses of hemoperfusion were performed using the Desept 150 column (polymer sorption device containing a bimodal hemosorbent based on styrene-divinylbenzene copolymer for the selective removal of bacterial endotoxins (lipopolysaccharides) and cytokines. Decept columns are approved for use in medical practice in Russia: Patents RU No. 2422160 and No. 89131, RU No. RZN 2013/714), with a filtrate volume of 18,000–39,600 ml, anticoagulant ACD-A solution, heparin 2.5–15 thousand IU, and 0.9% sodium chloride 500 ml. Additionally, pleural drainage was performed due to hydrothorax.

**Figure 1:**
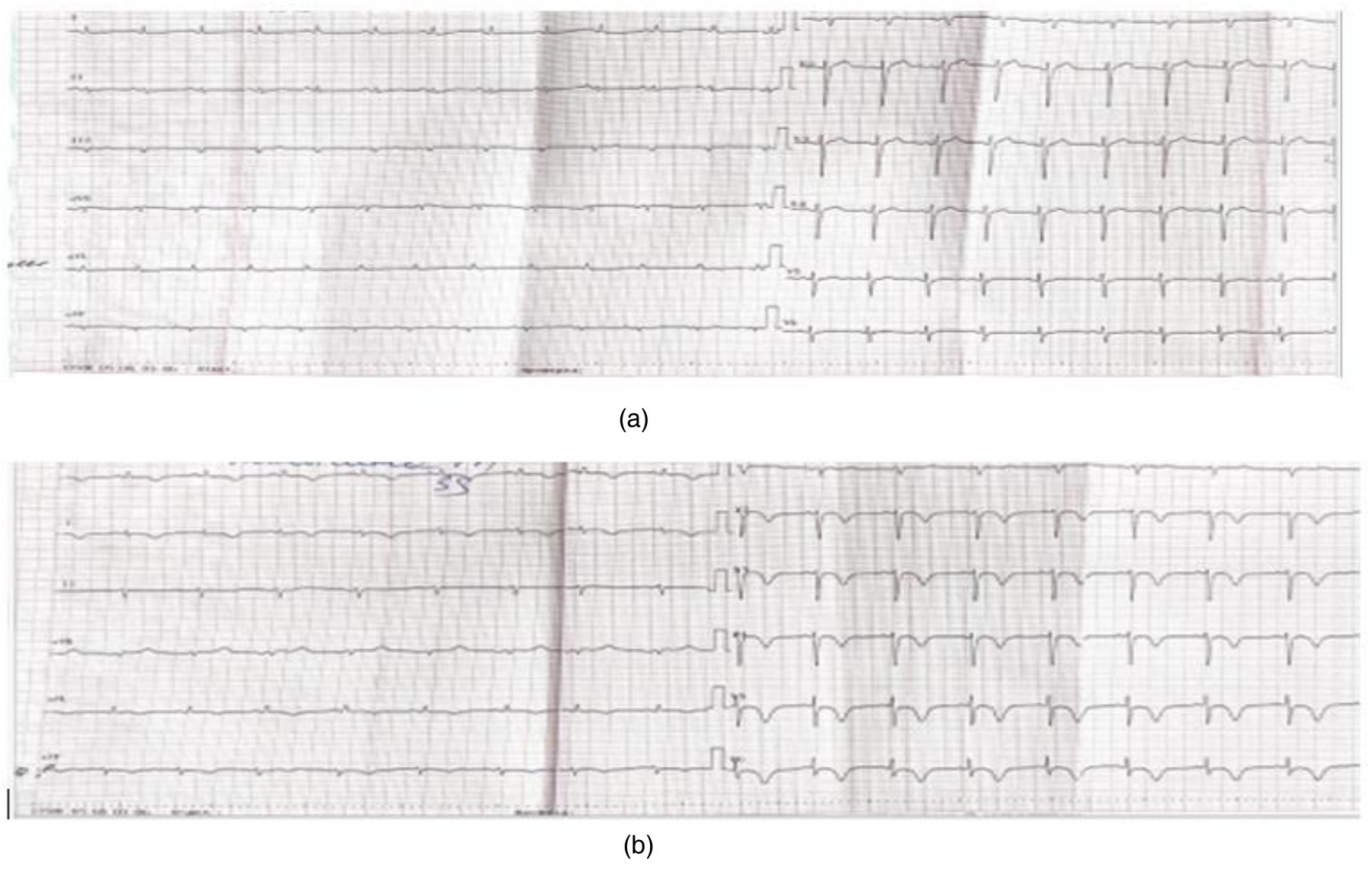
**(a)** (Pre-operative) twelve-lead electrocardiogram dated October 28, 2024. Sinus rhythm, 71 beats per minute, left axis deviation, reduced R-wave amplitude in all leads, and no evidence of acute ischemic changes. **(b)** (Post-operative twelve-lead electrocardiogram dated October 29, 2024. Sinus rhythm, 71 beats per minute, left axis deviation, reduced R-wave amplitude in all leads, T-wave inversion leads I, II, aVF, and V2-V6, abd ST-segment depression in leads V5-V6 up to 1 mm. QTc 478 ms.

On the ECG dated October 28, 2024, sinus rhythm with a heart rate of 71 beats per minute was recorded. Compared to the ECG from October 25, 2024, there was an inversion of the T wave in leads I, II, aVF, aVL, and V2-V6, as well as ST-segment depression in leads V5-V6 up to 1 mm. The QTc interval was 478 ms (Figures 1a,b).

**Figure 2:**
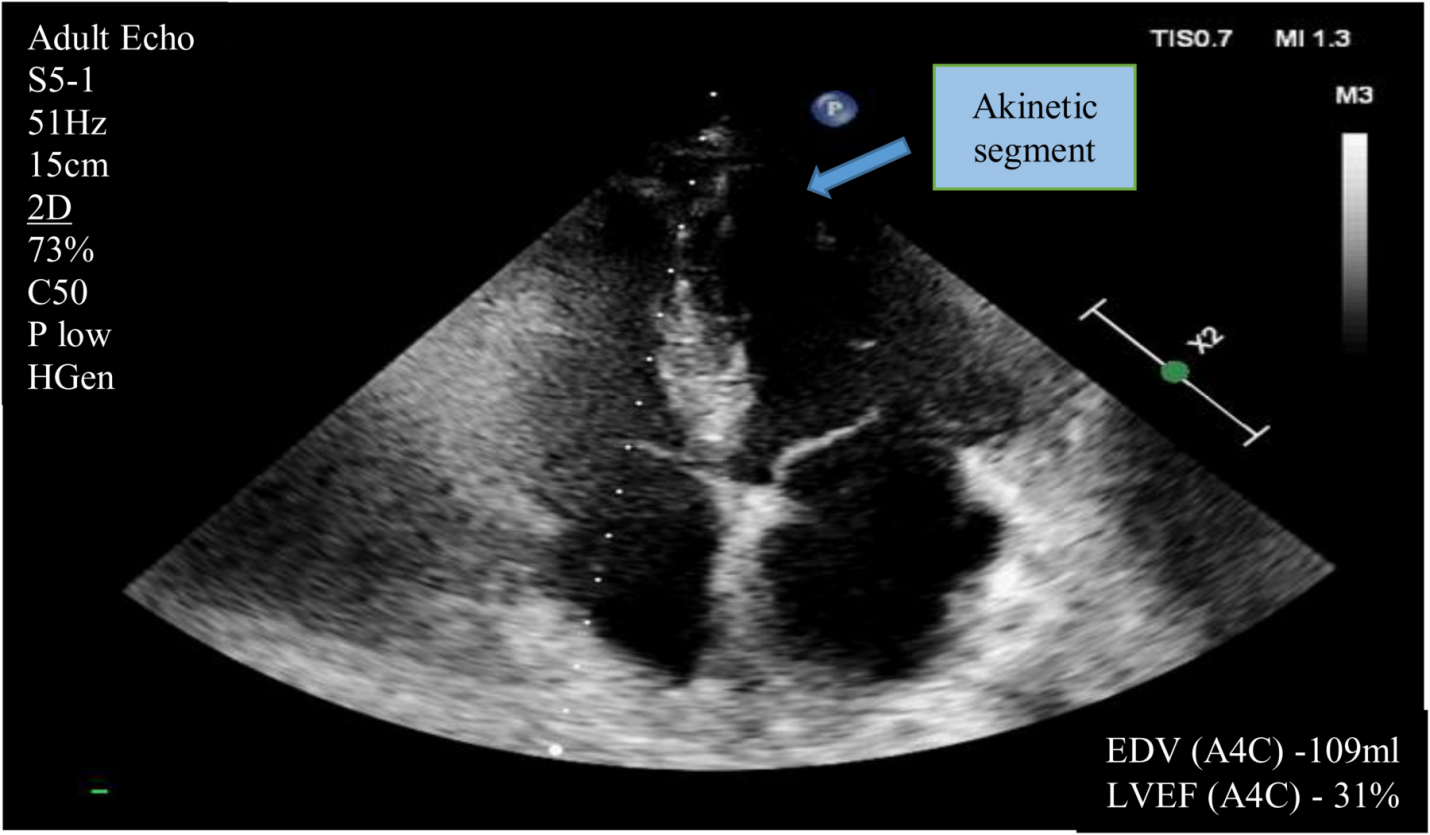
Transthoracic echocardiography (apical four-chamber view) demonstrating akinesis in the mid and apical levels of the anteroseptal segments and the posterolateral apical segments. Hypokinesis of the anteroseptal basal segments, and the posterolateral mid segments of the left ventricle. Also, the left ventricular ejection fraction measured using Simpson's method. (EF- Ejection fraction; EDV- End Distolic Volume.)

According to the echocardiography performed on October 28, 2024, there was a reduction in left ventricular ejection fraction (LVEF) to 31%–35% by Simpson's method. Akinesis of the anteroseptal segments at the mid and apical levels, as well as the posterolateral apical segments, was observed. Hypokinesis of the anteroseptal basal segments and the posterolateral mid segments of the left ventricle was also noted (prior to surgery, the LVEF was 63% with no areas of impaired regional wall motion) (Figure 2). Serial assessments revealed an increase in NT-proBNP levels to 8,864 pg/ml and troponin I levels to 129.4 ng/L (reference values <100 ng/L). October 28, 2024 NT-proBNP levels to 21,779 pg/ml and troponin I levels to 53,7 ng/L (reference values <100 ng/L).

Given the unstable hemodynamics, absence of anginal pain, recurrent anemia (down to 64 g/L), and thrombocytopenia (down to 78 × 10^9/L), a decision was made to perform coronary angiography after stabilization of the patient's condition and normalization of laboratory parameters. Cardiotropic therapy was administered, aimed at treating heart failure (ACE inhibitors, beta-blockers, aldosterone antagonists, if-channel inhibitors).

**Figure 3:**
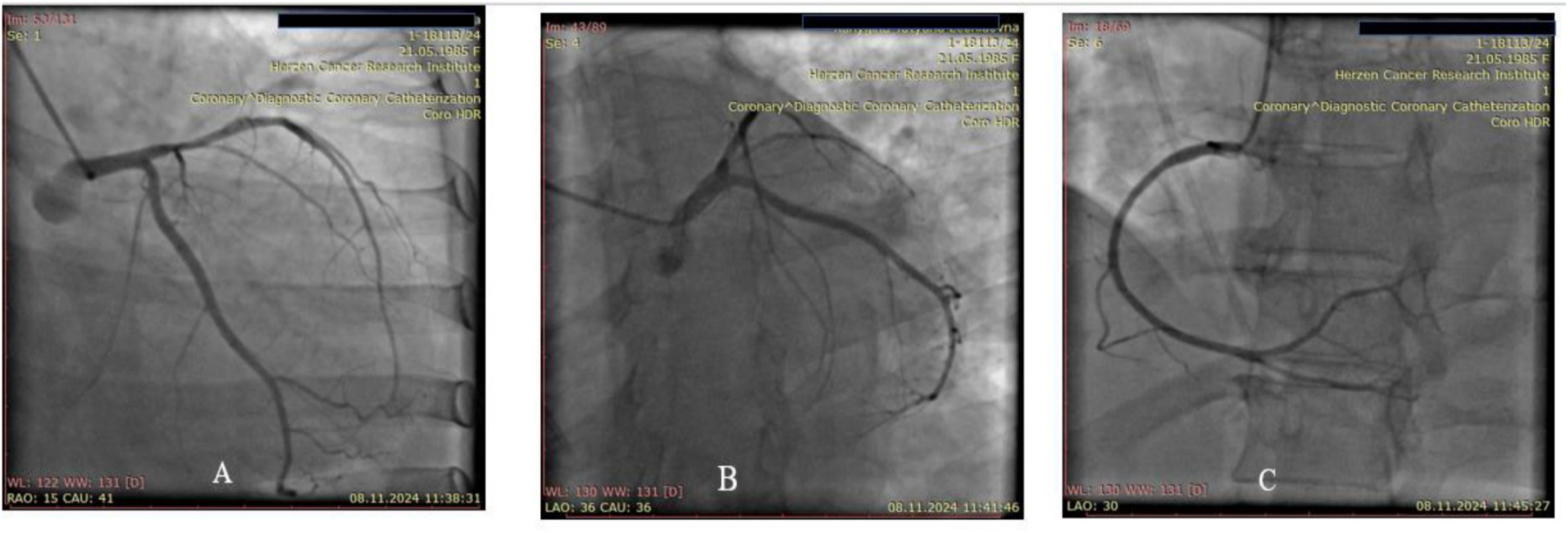
**(A)** Left coronary artery, posterior view. **(B)** Left coronary artery, spider view. **(C)** Right coronary artery, anterior oblique view.

On November 8, 2024., diagnostic coronary angiography was performed—no signs of coronary artery disease were detected (Figures 3A–C).

Thus, taking into account the changes observed on ECG, echocardiography, the dynamics of specific cardiac biomarkers, and the absence of stenotic lesions in the coronary arteries, the patient was diagnosed with Takotsubo syndrome according to the TCM criteria, described in International Expert Consensus Document on Takotsubo Syndrome 2018г (21). The patient was discharged in stable condition with recommendations for pharmacotherapy (according to Focused Update of the 2021 European Society of Cardiology (ESC) Guidelines for the diagnosis and treatment of acute and chronic heart failure 2023г (22): Valsartan/Sacubitril 12.5 mg twice daily, Bisoprolol 2.5 mg once daily, Ivabradine 5 mg twice daily, Spironolactone 100 mg once daily, and Dapagliflozin 10 mg once daily.

**Figure 4:**
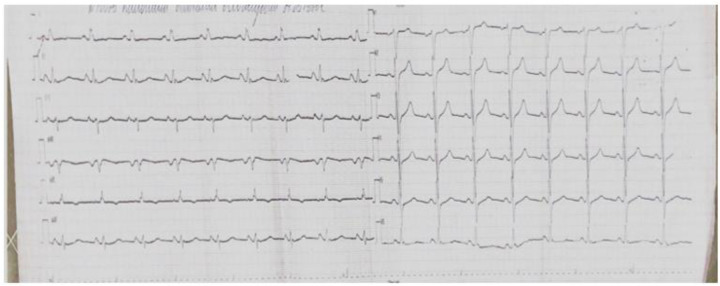
A 12—lead ECG taken on November 21, 2024—a positive trend: the ST segment has returned to the baseline, and the T wave has become positive.

According to the follow-up echocardiography dated November 26, 2024, the LVEF recovered to 51% by Simpson's method, with persistent mild hypokinesis of the anterior segment at the apical level and the anteroseptal segments at the basal and mid levels. On the ECG dated November 21, 2024, positive dynamics were noted: the ST segment returned to the isoelectric line, and the T wave became positive (Figure 4).

Additionally, a decrease in NT-proBNP levels from 27,779 to 2,794 pg/ml and in troponin I levels from 129 to 22.9 ng/L was observed.

## Discussion

In Takotsubo cardiomyopathy, excessive catecholamine production triggered by severe psychological or physical stress causes left ventricular dilation. In 75%–80% of patients, this occurs in the apical region of the left ventricle, creating the classic appearance of Takotsubo cardiomyopathy, resembling Japanese octopus traps. In 10%–20% of patients, dilation occurs in the mid-ventricular segments. Rarely, basal, biventricular, or focal ballooning has been observed (23). Ventricular ballooning leads to transient ST-segment elevation on ECG, elevated levels of troponin and B-type natriuretic peptide (BNP), and reduced ejection fraction, resulting in acute systolic heart failure (7). This mimics acute coronary syndrome (ACS) (ST-segment elevation myocardial infarction) or unstable angina. The most common symptoms are chest pain, dyspnea, or syncope (75.9%, 46.9%, and 7.7%, respectively), according to the International Takotsubo Registry (7). Uncertainties remain regarding its pathophysiology, diagnosis, and treatment. A recent prospective study from Japan found that among 43 patients with Takotsubo cardiomyopathy, 52% had physical triggers, 31% had emotional triggers, and 17% had no identifiable triggers (24).

Diagnosis «Сancer» undoubtedly affects the emotional and psychological state of the patient and causes several physical pathological changes in the body that can be a trigger for the development of TTS. Moreover, malignancy, some cancer treatments (5-FU, VEGFi), and the stress associated with the diagnosis, investigations, and treatment are recognized triggers or predisposing factors for TTS (25).

In our clinical case, we describe the development of TCM in a young female patient 39 years old with metastatic cancer post-emergency surgery without prior cardiac history. This distinguishes our clinical case from others typical TCM patients.

Based on the published literature, about 90% of TTS patients are women with a mean age of 67–70 years.

The patient in our clinical case received Bevacizumab, 5-fluorouracil, Irinotecan, Capecitabine and Oxaliplatin therapy in the past year. PCT from different groups can cause heart failure, however, no signs of systolic dysfunction of the left ventricle were detected according to echocardiography before surgery. Thus, the contribution of these chemotherapeutic drugs to the development of TCM in our case is unlikely. And that makes the contribution of surgical intervention more likely.

We have identified several cases that demonstrate the role of surgical intervention in the TCM development.

In Romania, a case was published of a 44-year-old patient who developed Takotsubo cardiomyopathy after cytoreductive surgery and hyperthermic intraperitoneal chemotherapy (HIPEC) for recurrent colon cancer (26). In this case, HIPEC appears to have played a role in the onset of Takotsubo cardiomyopathy.

Clinical case has been reported in the global literature involving an 80-year-old patient from Cajamarca, Peru, with blunt abdominal trauma and Chilaiditi syndrome. She was hospitalized for surgery but developed a complication resembling acute coronary syndrome (ACS) in the postoperative period, which was later diagnosed as TCM (27). Additionally, a similar postoperative complication occurred in a 50-year-old patient following surgical treatment of a hepatic hydatid cyst (28). There are also reports of TCM developing after liver transplantation (29) and after surgical treatment of small bowel obstruction (30). Some cases of Takotsubo cardiomyopathy have occurred after cardiac valve surgery. Vazhev and Stoev (31) published a case of Takotsubo cardiomyopathy developing after aortic and mitral valve replacement. Orthopedic surgeries can also be complicated by stress-induced cardiomyopathy. Authors have reported cases of TCM following bilateral simultaneous total knee arthroplasty (32) and after spinal surgery (33, 34).

## Conclusion

Takotsubo cardiomyopathy is an important differential diagnosis in patients presenting with symptoms of acute coronary syndrome (ACS), particularly in the context of a recent stressful event and the absence of obstructive coronary artery disease. The trigger may not only be psychological stress but also physical stress, including surgical interventions and their complications. Early diagnosis and appropriate treatment are essential to prevent potential complications and ensure a favorable outcome.

Clinicians should maintain a high index of suspicion for TCM in cancer patients undergoing emergency surgery, particularly with infectious complications or multiorgan failure.

## Data Availability

The original contributions presented in the study are included in the article/Supplementary Material, further inquiries can be directed to the corresponding author.
